# Expression of parathyroid hormone related protein (PTHRP) in ameloblastomas

**DOI:** 10.4317/jced.54222

**Published:** 2018-02-01

**Authors:** Rafael Zeballos, Ronell Bologna-Molina, Vanesa Pereira-Prado, Mariana Villarroel-Dorrego

**Affiliations:** 1 Hospital General del Oeste, Caracas, Venezuela; 2 Molecular Pathology Area, Faculty of Dentistry, Universidad de la República, Uruguay; 3 Oral Histopathology Laboratory, Universidad Central de Venezuela, Venezuela

## Abstract

**Background:**

Presence of parathyroid hormone related protein (PTHrP) might suggest that ameloblastomas recapitulate features of the enamel epithelium and induce bone resorption, which would facilitate their growth and local invasion. The aim of this study was to determine the expression of PTHrP in ameloblastomas.

**Material and Methods:**

An observational research study was designed including 39 cases of histologically diagnosed ameloblastomas (39 out of 42 patients gave consent for the use of their medical records and all data required for this study). Gender, age, tumor location, histological type and subtype of the tumor were recorded and PTHrP expression was determined by indirect immunohistochemistry using monoclonal anti-human PTHrP (1D1 / Santa Cruz Biotechnology). Protein expression and intensity were evaluated under light microscope and finally data recorded and statistically analyzed. This research was approved by the Caracas West General Hospital review board.

**Results:**

39 cases of ameloblastomas were evenly distributed between genders (49% male and 51% female) with a mean age of 33 ± 3.53 years, mainly affecting the posterior mandible. 20 cases (51.28%) showed positive cytoplasmic immunoreactivity to PTHrP. 8 out of 15 cases of solid/multicystic ameloblastomas and 12 out of 23 cases of unicystic ameloblastomas were PTHrP positive. Intense expression of PTHrP was observed in 4 unicystic ameloblastomas (all luminal subtype) and in 5 cases of conventional ameloblastomas.

**Conclusions:**

In the present study PTHrP expression in solid multicystic and unicystic ameloblastoma suggests its possible function in the biological behavior of the tumor. More studies are needed in order to determine the possible role of this protein related to bone invasion processes.

** Key words:**Parathyroid hormone related protein, PTHrP, ameloblastoma, bone.

## Introduction

Ameloblastomas are locally aggressive benign odontogenic tumors derived from odontogenic epithelium. There is an intimate relationship between the neoplastic cells and bone surrounding. However, there are not enough studies to establish the invasive nature of ameloblastomas. Some mechanisms by which ameloblastomas have a high growth rate and bone invasion may include factors related to the cell cycle and the tumor-bone interface (1-3). Overexpression of growth factors, apoptotic proteins, interleukins, cell adhesion proteins and matrix metalloproteinases have been observed in ameloblastomas (2-9).

Parathyroid hormone related protein (PTHrP) was originally described as a causal factor of humoral hypercalcemia of malignancy (10). It shares a common receptor with the parathyroid hormone (PTH) called PTH/PTHrP receptor, which is a class B G-protein coupled receptor (11). PTHrP comprised from 141 to 173 amino acids, due to alternative mRNA splicing, acting in a paracrine/autocrine fashion and intervening in regulation of cartilage and bone formation (12).

PTHrP is produced by various normal cells and tissues, including keratinocytes, heart and smooth muscle, endocrine cells, central nervous system (13) and dental organ (14-17). PTHrP also acts as an oncoprotein which is expressed on many malignant tumors, especially related to bone metastasis (18-20).

There is a strong evidence regarding the role of PTHrP in the activation of osteoclasts adjacent to the dental germ (17,21-23), that activation stimulates osteolysis which is necessary for dental eruption. PTHrP may play a similar role in ameloblastomas. The purpose of this study was to determine expression of PTHrP in ameloblastomas, which could simulate the process of an erupting tooth achieving tumor invasion of surrounding bone.

## Material and Methods

-Samples

39 cases of histologically diagnosed as ameloblastomas according to criteria of World Health Organization and International Agency for Cancer Research were included. 39 out of 42 patients gave consent for the use of their medical records and all data required for this study. Medical records were evaluated and paraffin blocks were selected for subsequent immunohistochemical analysis. The sample was derived from a population of patients, who attend the Oral and Maxillofacial Surgery Department at the West General Hospital, Caracas, Venezuela, from January 1, 2000 to December 31, 2004. All samples were obtained by incisional biopsy and the final histopathological diagnosis was confirmed with the surgical specimen. This research was approved by the Caracas West General Hospital ethical review board.

-Immunohistochemistry and microscopic evaluation of PTHrP

Indirect immunohistochemistry was performed. Tissues embedded in paraffin blocks were sectioned 4μm thick with a microtome. Three cuts were made per sample and placed on a glass slide previously embedded with poly-L-lysine. For antigen retrieval, samples were immersed in buffer 0.1 M sodium citrate and heated in microwave for 20 minutes. Tissues were then incubated with the primary antibody human monoclonal anti-PTHrP (Santa Cruz Biotechnology, San Francisco California, USA) at a dilution of 1:10 for 1 hour, washed and finally incubated with the secondary antibody (DAKO EnVision). The development of the reaction was performed using diaminobenzidine (DAB) and counterstained with Mayer’s hematoxylin. Sections of parathyroid adenoma were used as positive control, and for negative control, PBS was applied to substitute the primary antibodies.

PTHrP expression was evaluated under light microscopy. Immunostaining was described using a numeral scale from zero (0) to three (3), whereas 0 was negative, 1 was a weak immunostaining when neoplastic epithelial cells showed no uniform pale brown coloration, 2 for moderate immunostaining when the cells showed a brown discoloration even more intense but not in all of the cell islands, and then, a score of 3 was given to cases in which the intense brown staining was observed in all or most epithelial islands evaluated (24).

-Quantified variables

Gender, age, tumor location, histological type of ameloblastomas, expression and intensity of PTHrP were recorded. Data was analyzed using descriptive statistics. Presence or absence of PTHrP was quantitatively analyzed by number of cases and the intensity of the expression was qualitatively analyzed.

## Results

-Gender and age of the study population

Gender and age data are summarized in  Table 1. Lesions were evenly distributed between genders, 19 cases (49%) male and 20 cases (51%) female. The minimum age was 10 years and the maximum 67 years, with a mean age of 33 ± 3.53 years.

**Table 1:**
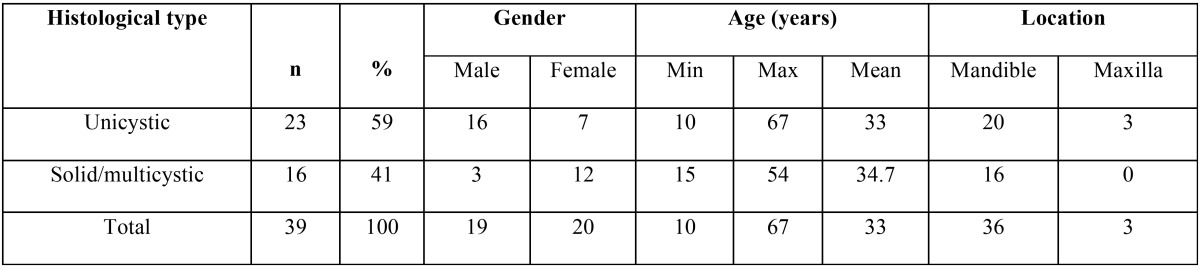
Ameloblastoma distribution by gender, age and location.

-Location of the tumor and histopathological classification

Three cases occurred in maxilla (8.33%) and 36 in the mandible (91.66%), mainly on the posterior zone ( Table 1). Regarding histological type, 23 cases (58.97%) were unicystic ameloblastomas (14 luminal and 9 mural), 16 cases (38.46%) multicystic-solid (conventional) ameloblastomas (6 plexiform, 2 acanthomatosous, 7 follicular, 1desmoplastic).

-Radiological image and tumor size

All unicystic ameloblastomas showed an unilocular radiographic image, while solid ameloblastomas showed a multilobulated image, with the exception of one case that showed an unilocular image. The mean tumor size was 4.5cm with a range of 1 to 6 cm.

-PTHrP expression

**Figure 1:**
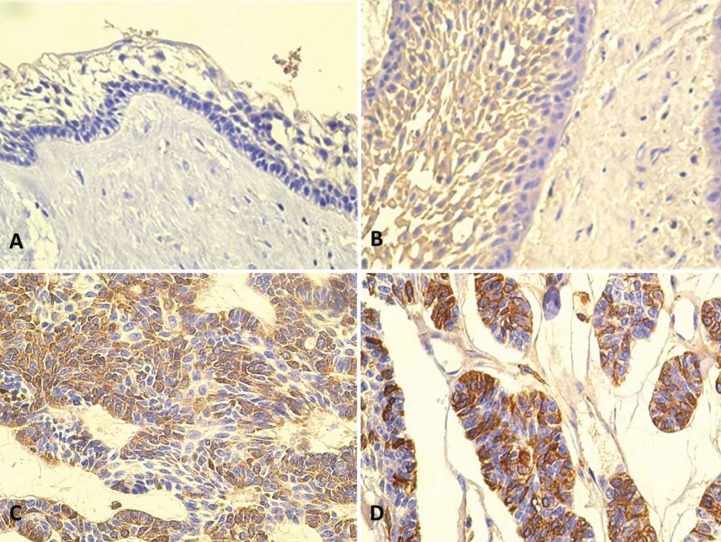
Microphotographs (optical microscope image 40X) of histological expression of PTHrP in ameloblastomas. Negative PTHrP expression in unicystic ameloblastoma (A), weak immunostaining for PTHrP expression (B), moderate PTHrP expression in solid/multicystic ameloblastoma(C) and intensePTHrP+ cells (D).

Out of 39 cases of ameloblastomas studied, 20 tumors (51%) showed positive cytoplasmic PTHrP expression, while 19 cases (49%) were negative. 9 cases of PTHrP-positive ameloblastomas showed an intense expression of the protein, 5 cases of moderate intensity and 6 cases showed a light expression (Fig. 1).

Interestingly, positive immunostaining was distributed among 8 cases of solid/multicystic (conventional) ameloblastomas (40% of positive cases) and 12 cases of unicystic ameloblastomas (60% of positive tumors). Intense expression of PTHrP was observed in 4 unicystic ameloblastomas (all luminal) and 5 cases of solid/multicystic ameloblastomas. Moderate expression was observed in 4 unicystic ameloblastoma (1 case luminal and 3 cases mural) and 1 case of acanthomatous ameloblastoma. A weak PTHrP expression was observed in 4 unicystic ameloblastoma (2 luminal) and in 2 cases of plexiform ameloblastomas ( Table 2). In some cases, cells reminiscent of the stellate reticulum showed a tendency to be less positive than the basal epithelial cells.

**Table 2:**
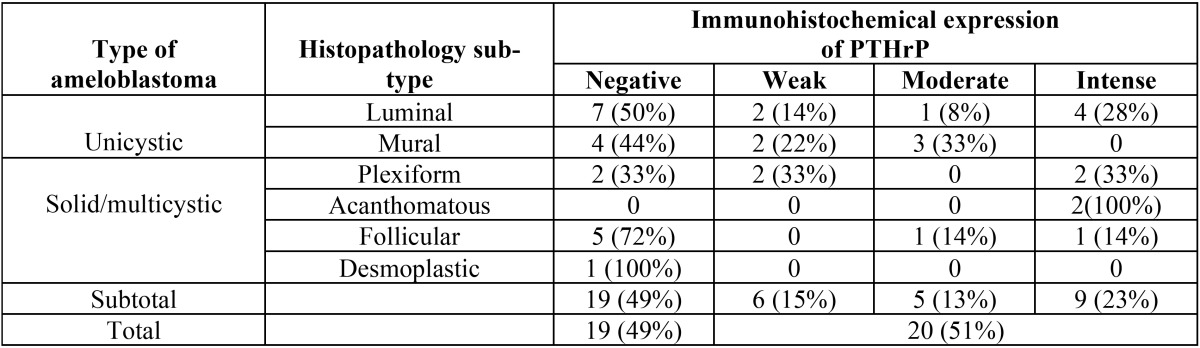
PTHrP expression according to ameloblastoma histopathology type and sub-type.

## Discussion

Recent advances about molecular pathogenesis of ameloblastoma determine that a great percentage show BRAF V600E mutations (25). Beyond genetic mutations, there are physiological mechanisms that are associated with the biological behavior of ameloblastoma.

The expression of PTHrP in ameloblastomas has been previously described by few studies (24-28). Results have suggested that PTHrP may be heavily involved in invasion and local bone penetration of ameloblastomas. Tumor may preserve ability of recapitulate features of the enamel epithelium, inducing bone resorption, such as during tooth eruption, via PTHrP expression.

Ameloblastomas are also able to express dental epithelium-specific transcription factors (29) which facilitate their growth and local invasion simulating dental eruption. Studies on mice have shown that PTHrP is expressed in the germinal epithelium of tooth enamel (14,15). While PTHrP is expressed in the reduced epithelium of the enamel organ, its primary receptor, PTH / PTHrP type I, is expressed in alveolar bone cells, dental cap and mesenchymal components of the developing tooth1 (6,30,31). PTHrP plays a significant role in osteoclast differentiation and alveolar bone resorption during the development of tooth germ and subsequent eruption pathway (14-17).

In bone physiology there is a balance between bone matrix production mediated by osteoblasts and bone resorption mediated by osteoclasts. TNF-α, IL-1β, vitamin D and PTHrP are potent calciotropic factors able to increases bone resorption and serum hypercalcemia (32). PTHrP may stimulate the differentiation and activation of the osteoclasts surrounding the tumor, allowing ameloblastomas to grow into adjacent bone and therefore increasing tumor invasion. Expression of PTHrP in ameloblastomas could be a potential predictor of tumor aggressiveness and prognosis, allowing treatment to be individually channeled by lesion behavior and not only by histological type or subtype. Moreover, it is suggested increase PTHrP blood level may be related to bone invasion and hypercalcemia in ameloblastomas (26).

In a previous study, Abdelsayed *et al.* (24) determined that intense PTHrP expression was observed only in conventional ameloblastomas, whereas unicystic ameloblastomas showed weaker levels of expression, hypothesizing that PTHrP expression is related to greater bone destruction in conventional ameloblastoma, and to the less aggressive behavior observed in unicystic tumors. In our study, there was no significant difference between unicystic and solid/multicystic (conventional) cases. This could be justified by the unique morphology of the unicystic ameloblastoma, which has a cystic lining epithelium formed by a few layers of basal and suprabasal epithelial cells, which showed a tendency to be positive. In contrast, conventional ameloblastoma shows a solid tumor morphology with large areas of stellate reticulum-like cells. In other words, we think that the proportion of the diverse types of epithelial cells, as well as the different mechanisms of growth in unicystic and conventional ameloblastoma, may influence the results of the positive index. These results suggest that PTHrP expression is involved in ameloblastoma invasive and destructive behavior, beyond the histological subtype.

One limitation of this study was the number of cases included, so it is suggested to carry out more studies with bigger casuistic to rule out the existence of a significant differences depending on the histological type. In our study, more than half of lining epithelium of uniquistic ameloblastomas were positive for PTHrP. Abdelsayed *et al.* (24) also demonstrated that PTHrP expression was seen in lining epithelium of unicystic ameloblastomas, as well as in the lining epithelium in dentigerous cysts exhibiting ameloblastomatous changes but not in those lacking those variations. PTHrP might become a valuable aid in the diagnosis of ameloblastomatous changes.

In the present study PTHrP expression in solid multicystic and unicystic ameloblastoma suggests its possible function in the biological behavior of the tumor. More studies are needed in order to determine the possible role of this protein related to bone invasion processes.
